# The domain swapping of human cystatin C induced by synchrotron radiation

**DOI:** 10.1038/s41598-019-44811-1

**Published:** 2019-06-12

**Authors:** Michal Taube, Zuzanna Pietralik, Aneta Szymanska, Kosma Szutkowski, Daniel Clemens, Anders Grubb, Maciej Kozak

**Affiliations:** 1 Department of Macromolecular Physics, Adam Mickiewicz University in Poznań, Uniwersytetu Poznańskiego 2, 61-614 Poznań, Poland; 2 Joint Laboratory for SAXS Studies, Adam Mickiewicz University in Poznań, Uniwersytetu Poznańskiego 2, 61-614 Poznań, Poland; 3 Faculty of Chemistry, University of Gdańsk, Wita Stwosza 63, 80-308 Gdańsk, Poland; 4 NanoBioMedical Centre at Adam Mickiewicz University in Poznań, Wszechnicy Piastowskiej 3, 61-614 Poznań, Poland; 5 Helmholtz-Zentrum Berlin für Materialien und Energie Lise-Meitner-Campus Hahn-Meitner-Platz 1, 14109 Berlin, Germany; 6 Department of Clinical Chemistry, Lund University Hospital, S-22185 Lund, Sweden

**Keywords:** Biophysics, SAXS

## Abstract

Domain swapping is observed for many proteins with flexible conformations. This phenomenon is often associated with the development of conformational diseases. Importantly, domain swapping has been observed for human cystatin C (HCC), a protein capable of forming amyloid deposits in brain arteries. In this study, the ability of short exposure to high-intensity X-ray radiation to induce domain swapping in solutions of several HCC variants (wild-type HCC and V57G, V57D, V57N, V57P, and L68V mutants) was determined. The study was conducted using time-resolved small-angle X-ray scattering (TR-SAXS) synchrotron radiation. The protein samples were also analysed using small-angle neutron scattering and NMR diffusometry. Exposing HCC to synchrotron radiation (over 50 ms) led to a gradual increase in the dimeric fraction, and for exposures longer than 150 ms, the oligomer fraction was dominant. In contrast, the non-irradiated protein solutions, apart from the V57P variant, were predominantly monomeric (e.g., V57G) or in monomer/dimer equilibrium. This work might represent the first observation of domain swapping induced by high-intensity X-rays.

## Introduction

Domain swapping has frequently been observed in several proteins associated with conformational diseases^1,2^. This phenomenon is also associated with the development of neoplastic diseases (e.g., B-cell lymphoma-2-associated X (Bax) protein^3^). A good example of proteins involved in the development of neurodegenerative diseases such as human spongiform encephalopathies is the prion protein, for which the formation of dimers is governed by domain swapping^2^.

**Figure 1:**
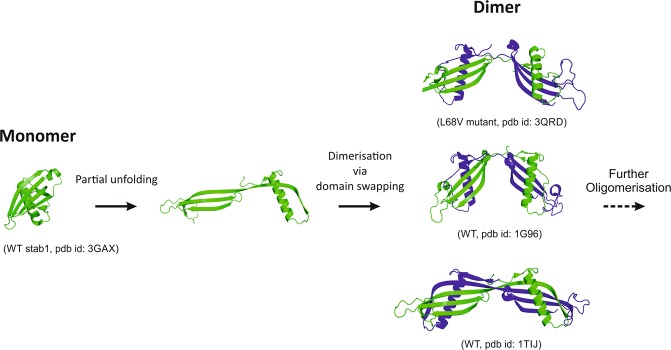
Structure of human cystatin C and schematic representation of the domain swapping process and formation of dimers from monomers. Structure of the covalently stabilized HCC monomer (PDB code: 3GAX)^30^. Structures of three HCC dimers with different conformations (from the top: HCC L68V, PDB code: 3QRD; native form 1 (cubic polymorph) HCC, PDB code: 1G96; and native form 2 (tetragonal polymorph) HCC, PDB code: 1TIJ)^4,6^.

In human cystatin C (HCC), domain swapping is closely related to the susceptibility of this protein to oligomerization, which leads to the formation of amyloid deposits and brain haemorrhage^4–7^. The domain swapping mechanism observed for HCC (shown in Fig. 1) is based upon partial unfolding of the HCC monomer (dissociation of the N-terminal region containing the core α-helix) and its subsequent association with another HCC molecule. As a result, HCC dimers are formed that also recreate the general HCC fold due to interchangeable binding of neighbouring analogous domains^4^. This mechanism is also thought to be involved in the formation of higher-order HCC oligomers and further fibril formation^8,9^. Recently, an oligomerization mechanism that does not involve domain swapping was proposed for the V57N variant of HCC^10^.

Proteins capable of swapping domains are hypothesized to share some common structural elements. For instance, so-called hinge loops are conformationally constrained flexible turns with unfavourable torsion angles or that contain particular amino acid residues, such as proline^11^. In cystatin C, loop L1 (residues Q^55^IVAG^59^) is thought to act as such a molecular hinge, connecting two HCC fragments that undergo exchange during domain swapping, namely, the N-terminal β1-α-β2 and C-terminal β2-AS-β4-L2-β5 fragments. Conformational tensions in this fragment are centred at valine 57, which is located near the apex of the turn. Substituting this residue with residues expected to stabilize the loop conformation (e.g., Asp, Asn and Gly) yields HCC variants with increased monomeric stability, as observed in solution and partially in the solid (crystal) state (HCC V57N and V57G). The third variant, HCC V57D, despite being relatively stable in solution, formed a dimeric structure during crystallization trials, similar to the behaviour of the wild-type protein. The pivotal role of L1 as a hinge loop during HCC domain swapping was confirmed via loop broadening through the introduction of a destabilizing proline residue at Val57. The resulting mutant was not only predominantly dimeric after overexpression and purification but also showed an increased propensity towards further oligomerization and aggregation in many tests^12^.

In macromolecular studies, radiation damage caused by synchrotron radiation is particularly important problem in protein crystallography. Numerous attempts have been made to determine optimal radiation doses and data collection strategies to prevent sample damage^13–15^. Recently, a dose-limiting data collection strategy for serial synchrotron measurements of microcrystalline protein samples has been proposed to reduce the effects of radiation damage^16^. Notably, synchrotron radiation causes not only local structural changes in the crystal (e.g., decarboxylation of aspartic and glutamic acid residues or radiolysis of disulfide bridges)^17,18^ but also a global increase in temperature factors, which is related to increasing disorder in the crystal after irradiation^19,20^. To the best of our knowledge, global long-range structural changes in the crystal, such as domain swapping induced by X-ray irradiation, have yet to be observed, which is not surprising because such changes would most likely destroy the crystal lattice. However, these changes cannot be excluded in solution, such as during biological small-angle X-ray scattering (BioSAXS) experiments. Just as in crystals, synchrotron radiation photons can induce the formation of free radicals or cause the breakdown of disulfide bonds in solution, which can result in protein aggregation^21,22^. As a result, SAXS data obtained from measurements of an aggregated protein sample (polydisperse) are difficult or impossible to interpret. Most often, the problem of radiation damage is discussed with respect to how this damage can be reduced^21,23,24^. Aside from protein aggregation or potential radiolysis, the effect of radiation on protein conformation has not been extensively analysed.

The aim of our study was to characterize the influence of synchrotron irradiation on the fast conformational changes of HCC in solution. In particular, the most important issue was to establish whether exposure of a protein solution to synchrotron X-ray irradiation would be sufficient to induce domain swapping between HCC molecules. The studies were performed on wild-type HCC (wt-HCC) and selected point mutants at position 57 (i.e., the hinge loop region: V57N, V57G, V57P, V57D) or 68 (L68V). The L68Q mutation in cystatin C causes the dominantly inherited disease “Hereditary Cystatin C Amyloid Angiopathy”, with a very high mortality caused by brain haemorrhage. To exclude the spontaneous oligomerization of the HCC variants in solution without irradiation, the samples were also characterized using small-angle neutron scattering (SANS) and nuclear magnetic resonance (NMR) diffusion measurements.

## Materials and Methods

### Expression and purification of HCC variants

Oligonucleotides for site-directed mutagenesis and dNTPs were purchased from Oligo.pl (Warsaw, Poland) or Sigma Aldrich (Poland, Poznan). DNA sequencing was performed at the Laboratory for Nucleic Acid and Protein Detection (Pomeranian Science and Technology Park, Gdynia, Poland). Pfu polymerase was purchased from Fermentas. S-Sepharose, Superdex 75 PC 10/300, Superdex 75 PC 3.2/30 columns and Gel Filtration Low Molecular Weight Calibration Kits were from GE Healthcare (Warsaw, Poland). DNA purification kits were purchased from A&A Biotechnology (Gdansk, Poland). Unless otherwise specified, all reagents were of molecular biology or analytical grade.

### Bacterial strains, plasmids and mutagenesis

The plasmid pHD313, which encodes HCC fused to the OmpA signal peptide for periplasmic expression, was used for the construction of new cystatin C variants^25^. The designed point mutations were introduced via site-directed mutagenesis using Pfu polymerase according to the manufacturer’s instructions. DNA was amplified in *E. coli* DH5α competent cells and purified using a DNA purification kit. Mutagenesis was confirmed by DNA sequencing.

### Protein expression and purification

Wild-type cystatin C and its variants were obtained using the protocol described by Szymanska *et al*. with minor modifications^12^. Proteins were produced in *E. coli* C41(DE3). Protein expression was induced thermally at an OD_600_ value of approximately 0.7–0.8 by quickly raising the temperature to 42 °C for 30 min. The temperature was then lowered to 40 °C, and the culturing was continued for 3 h. The bacteria were then harvested by centrifugation. The pellet was resuspended in buffer containing 20 mM Tris, pH 7.5, 10% glycerol, and 1 mM benzamidinium chloride (20 ml/0.5 L of culture) and flash-frozen in liquid nitrogen. Finally, the samples were slowly thawed on ice and then flash-frozen again.

Crude proteins were isolated using a modified cold osmotic shock procedure^26^. The supernatant from the freeze-thaw steps, containing significant amounts of the overexpressed proteins, was collected in addition to the fractions typically obtained in this method (saccharose and periplasmic). Proteins were purified by ion-exchange chromatography on S-Sepharose and eluted with a linearly increasing salt gradient (0 to 0.5 M NaCl in 20 mM Tris, 1 mM benzamidinium chloride, pH 7.5). Fractions containing the target proteins were collected, extensively dialyzed against 10 mM ammonium bicarbonate, pH 8.0, and lyophilized. Finally, proteins were purified to homogeneity on an FPLC Superdex 75 PC 10/300 column in 25 mM ammonium bicarbonate, pH 8.0. Pure protein fractions were lyophilized and stored at −20 °C. Protein purity was confirmed using electrophoresis, gel filtration and mass spectrometry.

### Time-resolved small-angle X-ray scattering (TR-SAXS)

TR-SAXS experiments were performed at the EMBL P12 beamline on the PETRA III storage ring synchrotron at DESY in Hamburg (Germany). The X-ray photon flux on the sample was 10^12^ photons/s. Just before measurements, lyophilized protein samples (wt-HCC, V57N, V57G, V57P, V57D and L68V) were dissolved in standard phosphate buffered saline (PBS), pH 7.4, and the concentrations were estimated using the extinction coefficient at 280 nm. The protein concentrations of the HCC variants used in the TR-SAXS studies ranged from 4.3 to 5.6 mg/ml. Protein solutions were loaded into the thermostatted cell and kept at 10 °C. In total, 20 consecutive 50-ms frames were recorded along the s-vector from 0.05 < *s* < 3.5 nm^−1^ (*s* = 4πsin(*θ*)/*λ*). Data were collected using synchrotron radiation (*λ* = 0.124 nm). The exposure time for each frame was 45 ms, while the read-out time was 5 ms (a total of 50 ms per exposure). Each HCC variant was measured three times independently using a hybrid photon counting detector, Pilatus 2 M, with an active sensor area (width × height) of 253.7 × 288.8 mm^2^ (DECTRIS AG, Switzerland). Data reduction and buffer subtraction were performed for each individual frame using the PRIMUS program from the ATSAS package^27,28^. The radius of gyration (*R*_*g*_) was calculated in AutoRG using the Guinier approximation formula: *I*(*s*) = *I*(0)e^[(*sR*^_*g*_^)2/3]^, valid at small s values (*s* < 1.3 *sR*_*g*_)^27,29^.

### SAXS data processing and analysis

HCC structures deposited in the PDB, 3GAX (monomer-stabilized HCC variant)^30^ and 1TIJ (domain-swapped HCC dimer)^6^, were used to calculate the theoretical scattering profiles and for fitting to the experimental data. SAXS data for the individual frames were used for singular value decomposition (SVD) and multivariate curve resolution alternating least squares (MCR-ALS) analysis^31^. Initial SVD analysis was performed using the SVDPLOT procedure implemented in Primus. A statistically significant number of singular vectors was estimated with a built-in Wald-Wolfowitz test. Final SVD analysis was performed in MATLAB R2014 using the svd function. Autocorrelation curves were calculated using the MATLAB autocorr function. The number of components was chosen to be the number of the autocorrelation functions of the eigenvectors U that differ from the noise. MCR-ALS analysis was carried out for the first five frames in the time range from 50 ms to 250 ms for a single SAXS measurement. The maximum s vector value used in the analysis was 2 nm^−1^ or 3 nm^−1^, depending on the HCC variant data quality. The analysis was performed in MATLAB using the MCR-ALS toolbox^32^.

### SAXS data collection using a laboratory X-ray source

SAXS studies of reference samples (wt-HCC and its variants) were performed using a laboratory SAXS/WAXS XEUSS 2.0 system (XENOCS, France). X-ray radiation (*λ* = 0.134 nm) was generated by the MetalJet microfocus X-ray source with a liquid metal (gallium alloy) target (Excillum AB, Sweden) and collimated using a Fox 3D Ga ultra-low divergence mirror. The HCC samples were measured using a low-noise flow cell and a PILATUS 3 R 1 M hybrid photon counting detector with an active sensor area (width × height) of 169 × 179 mm^2^ (DECTRIS AG, Switzerland). The sample-to-detector distance was 1200 mm, which in the high-flux mode of X-ray optics, covers the scattering vector range 0.08 < *s* < 4.7 nm^−1^. The X-ray photon flux on the sample was 2 × 10^8^ photons/s. For each sample, 15 frames (600 s/frame) were collected. The collected frames were checked for radiation damage and processed using FOXTROT^33,34^, and the buffer scattering was subtracted using PRIMUS^28^. The low-resolution parameters (*R*_*g*_, pair distance distribution functions) were then analysed from the SAXS data using the software described above.

### Small-angle neutron scattering (SANS)

The SANS studies of wt-HCC and its mutants were performed in the identical experimental conditions as previously were applied for covalently stabilised HCC trimers^35^. For the SANS experiments protein samples were prepared in 50 mM sodium phosphate/D_2_O, pD 7.0 with final protein concentration from 6.5–8 mg/ml. To remove any potential HCC aggregates, all samples were centrifuged at 13000 rpm before the measurements. Finally, protein samples were placed in quartz glass cuvettes at 15 °C. The SANS experiments were performed at the V16 SANS beamline at BER II reactor at the Helmholtz-Zentrum Berlin (Germany)^36^. During the SANS experiments two sample-to-detector distances were used: 1.7 m and 11.23 m corresponding to two collimation lengths: 6 m and 12 m. Scattering vector range covered by the experimental setup was: 0.65 < *s* < 6 nm^−1^ and 0.035 < *s < *0.79 nm^−1^, for the short and long camera setups respectively. The diaphragms at the collimator entrance were set to 40 × 40 mm^2^, while at the sample, the diaphragm had a diameter of 7.5 mm. Scattering patterns were recorded using 2D ^3^He detector with 100 × 100 cm^2^ area. Data processing and reduction were done using MANTID software^36,37^.

### Self-diffusion studied by pulsed gradient spin-echo (PGSE) NMR

The hydrodynamic radii of the proteins in the study were estimated from the self-diffusion coefficients obtained at 21 ± 0.1 °C and Stokes-Einstein relation *R*_*h*_ = k_B_*T*/6π*ηD* (k_B_ is the Boltzmann constant, *η* is the solvent viscosity and *D* is the self-diffusion coefficient). The experimental values of diffusion coefficients were obtained by Agilent DD2 14 T NMR spectrometer equipped with a dedicated diffusion probe (DOTY DSI-1374, max gradient pulse 28 T/m). The NMR pulse sequence in use was Dbppste (bipolar pulse pair-stimulated echo) with repetition delay 5 s, diffusion delay Δ = 50 ms, gradient pulse duration δ = 1 ms and gradient pulse amplitudes varied from 0 to 4 T/m. The values of self-diffusion coefficients *D* were extracted from the slope of the ln(*A*) vs *g*^2^ dependence where *A* denotes integral amplitudes extracted from the 0–4 ppm region in the NMR spectra and *g* are the subsequent amplitudes of the magnetic field gradient pulses. Then self-diffusion coefficients are obtained from the Stejskal-Tanner relation by fitting the linear dependence to ln(*A*) vs *g*^2^ and using the following relation $D=-slope/{\gamma }^{2}{\delta }^{2}({\rm{\Delta }}-1/3\delta )$, where *γ* is the gyromagnetic ratio for protons^38^.

## Results

**Figure 2:**
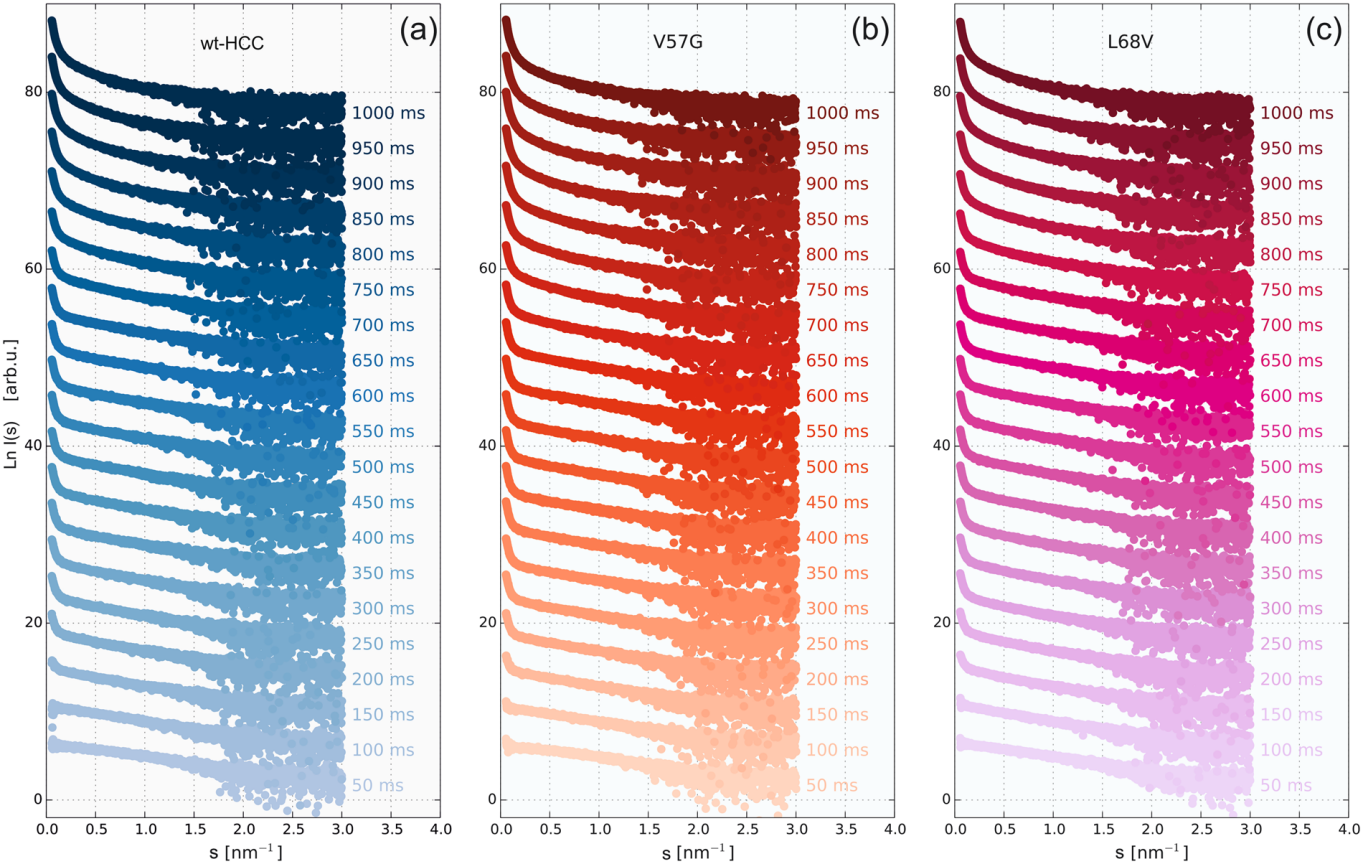
Exemplary SAXS data collected for wild-type HCC (**a**) and two HCC variants, V57G (**b**) and L68V (**c**).

The experiments described in this paper were performed in two stages. First, we characterized the radiation-induced oligomerization wt-HCC and its variants. These studies included the TR-SAXS experiments using synchrotron radiation. Second, the same protein variants were studied to verify their monodispersity (exclusive or dominant presence of the monomeric form of HCC in solution) using SANS, NMR diffusometry and SAXS, but under conditions that would not induce radiation damage in the samples. TR-SAXS experiments using synchrotron radiation were conducted without sample flow during measurements. Individual SAXS images were recorded with a 50-ms exposure time (including detector read-out time of approximately 5 ms). Subsequently, individual exposures were accumulated up to 2000 ms. For simplicity, in the presentation and later discussion of the results obtained, we will use exposure values that are multiples of a single exposure time (50 ms), neglecting the relatively short read-out time. Examples of full SAXS datasets for wt-HCC and two selected variants (V57N and L68V) are shown in Fig. 2 (TR-SAXS data for other HCC variants are presented in the Supplementary Information – Fig. S1).

The HCC variants selected for our studies were those in which loop L1 is conformationally stabilized by single-amino acid substitutions: V57N, V57D or V57G. These variants exhibit high resistance to dimerization and fibrillization^12^. Another HCC variant contains a mutation (L68V) within the hydrophobic core of the molecule. This variant exhibits a slightly increased dimerization tendency compared with wild-type HCC^39^. The last HCC variant used in this study (V57P) exhibits high conformational flexibility within the L1 loop and a high propensity for dimerization and oligomerization.

Preliminary qualitative analysis of the recorded SAXS curves already revealed different susceptibilities to dimerization and further gradual oligomerization of individual variants of cystatin C. In particular, the beginning of the SAXS curve (at low *s*-values) exhibits an increase in scattering intensity with exposure time, which is characteristic of gradual protein aggregation. Therefore, subsequent TR-SAXS data analysis was carried out at several stages.

**Figure 3:**
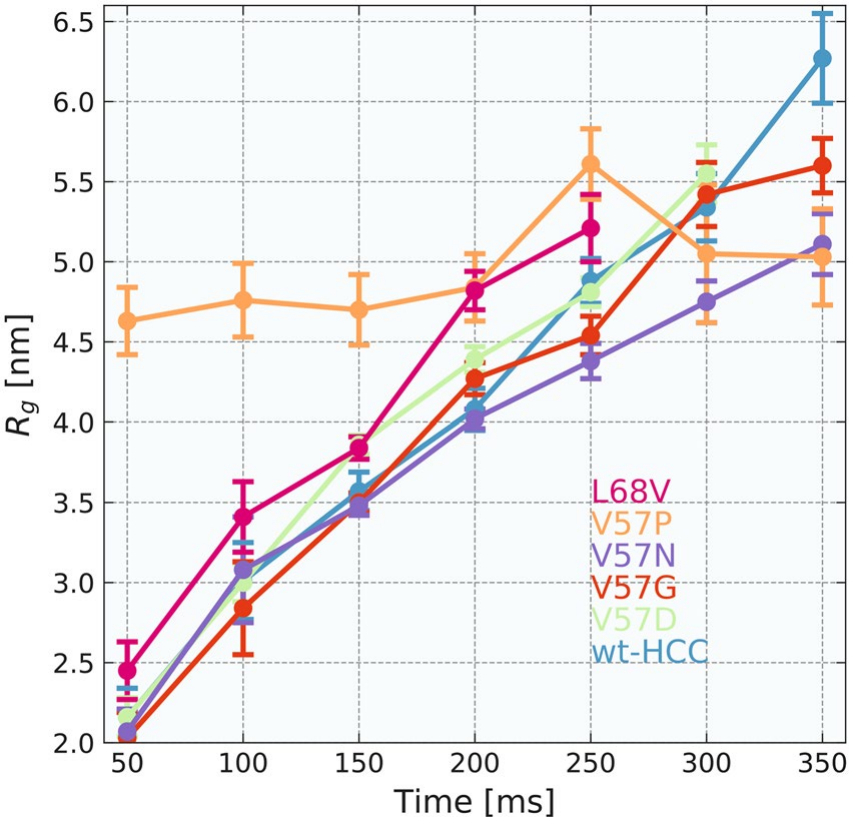
Radii of gyration, *R*_*g*_, calculated for wild-type human cystatin C and all HCC variants studied.

These stages are summarized and shown in Fig. 3 and Supplementary Table S1. In the first step of the analysis of the TR-SAXS data for the first 5 to 7 frames (for wt-HCC and for all HCC variants), recorded as a function of time, the radii of gyration, *R*_*g*_, were determined.

**Figure 4:**
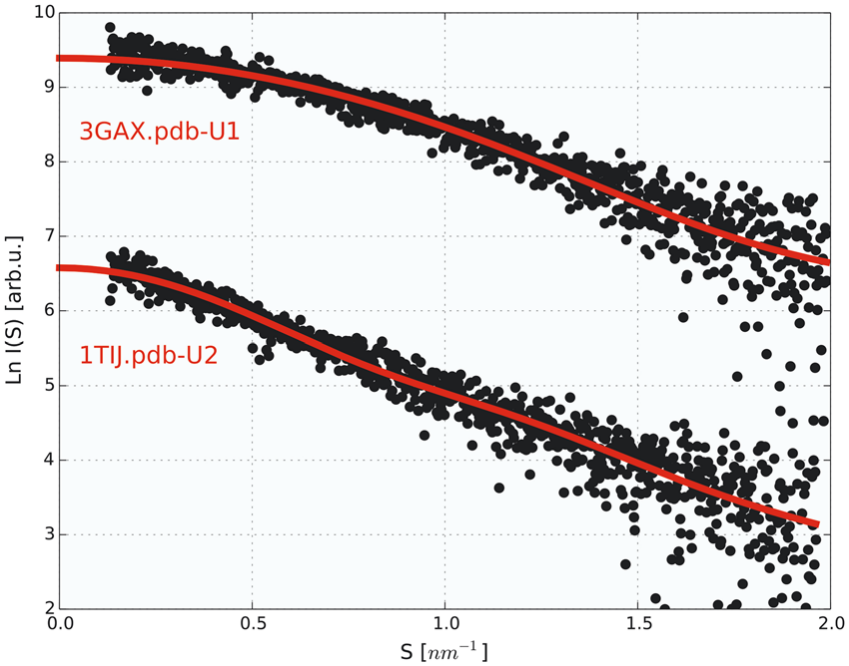
Comparison of SAXS data (wild-type HCC) derived from the MCR-ALS analysis with theoretical scattering curves calculated from the atomic structures of monomeric (PDB code: 3GAX) and dimeric (PDB code: 1TIJ) human cystatin C variants.

**Figure 5:**
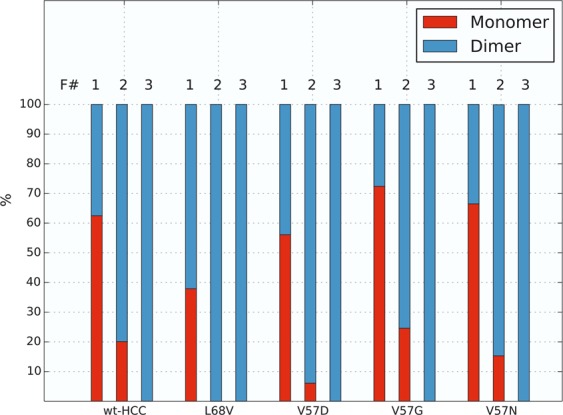
Normalized fractions of monomers and dimers calculated from the SAXS curves recorded for wild-type and mutant HCC variants (frames 1–3, after 50-, 100- and 150-ms exposure to synchrotron radiation).

The values of *R*_*g*_ values for the 50-ms exposure time ranged from 2.03 ± 0.16 to 2.45 ± 0.18 nm for wt-HCC and all HCC variants, except for V57P. These values indicate that during the first exposure to synchrotron radiation in almost all samples (except for V57P and L68V) the monomeric form of cystatin C dominated. In contrast, V57P showed a strong tendency to aggregate at the very beginning. *R*_*g*_ for this variant was 4.63 ± 0.21 nm, and during subsequent expositions, *R*_*g*_ stayed in the range of 4.70 ± 0.22–5.61 ± 0.22 nm. For wt-HCC and other HCC variants, we observed a gradual increase in *R*_*g*_ as a function of exposure time of the protein solution to synchrotron radiation. *R*_*g*_ calculated for the samples after 100 ms exposure reached values from 2.84 ± 0.29 nm (V57G) to 3.41 ± 0.22 nm (L68V), and after 200 ms exposure, *R*_*g*_ increased significantly to 4.02 ± 0.06 nm (V57N), 4.08 ± 0.13 nm (wt-HCC) or up to 4.82 ± 0.21 nm (L68V). These changes indicate gradual aggregation or oligomerization in the protein samples. To examine this phenomenon in detail, we analysed the fraction of HCC monomer, dimer and oligomer at the initial stages of the TR-SAXS experiment (50 to 150-ms exposure). First, the number of independent components contributing to the recorded data was estimated, and the original experimental scattering curves were subsequently decomposed into individual scattering curves corresponding to each component. The number of three independent components was estimated using the SVD method (see Figs S2 and S3 in the Supplementary Materials) from the 50 ms to 250 ms frames. MCR-ALS analysis was then used to extract scattering curves for the number of components estimated from SVD. Importantly, a similar method for SAXS data analysis using chemometric methods was successfully applied by Herranz-Trillo *et al*.^40^ to analyse the formation of multiple fibrillogenic species of insulin and the familial E46K variant of α-synuclein. In the approach to reconstruct the scattering data, in the first step the number of components in the system is estimated using SVD algorithm. The number of components is chosen to be the number of autocorrelation functions of eigenvectors U that differ from the noise. In the next step the MCR-ALS algorithm determined the scattering curves for the number of components estimated using SVD. MCR-ALS algorithm reconstructs the scattering curves for the individual components without the previous knowledge about the structure of the components in an iterative fashion. The only constrains are the non-negativity of the scattering curve values and that the system in closed, so the total concentration of components is equal to 1. Detailed description of the method is presented in Blobel *et al*.^31^. Scattering curves for the three independent components obtained from MCR-ALS analysis are presented in Fig. S4 in the Supplementary Materials. The first and second components were fitted well using the structures of monomeric and dimeric HCC, and the fits are presented in Fig. 4 and in Table S2. The third scattering curve represents a higher-order oligomeric component. Because of the low signal-to-noise ratio, the obtained fractional content for each component may be prone to error. Nevertheless, this result clearly showed that a transition from the monomeric to the domain-swapped dimeric form occurs during the first stage of oligomerization. To more quantitatively analyse the first stage of oligomerization, we next fitted the data for the first three exposure frames using a combination of the structures for monomeric and dimeric HCC. The reference atomic models for determining the contribution of various oligomeric forms of HCC to the scattering curve were the atomic structures of the HCC monomer (PDB code 3GAX) and HCC dimer (PDB code 1TIJ). We were able to establish that the SAXS curves recorded in the first 50 ms for the wt-HCC form and the other variants (except L68V and V57P) contained predominantly monomers. These samples contained from 56.1% (V57D) to almost 72.4% (V57G) monomeric fraction, while in the wt-HCC sample, the monomer fraction was 62.5%. The second component was the dimer (from 27.6 to 43.9%). A detailed summary of fractions ascribed to HCC monomer and dimer, made using the OLIGOMER program, is presented in Table 1 and in Figs 5 and S5 (Supplementary Materials).

**Table 1:** Monomer and dimer fractions of HCC variants calculated using OLIGOMER for frames 1 (exposure time: 50 ms), 2 (100 ms) and 3 (150 ms).

Cystatin C variant	Species	Frame 1 [%]	χ^2^	Frame 2 [%]	χ^2^	Frame 3 [%]	χ^2^
wt-HCC	Monomer	62.5	1.00	20.1	1.03	0	1.57
	Dimer	37.5		79.9		100	
L68V	Monomer	37.9	1.04	0	1.32	0	3.64
	Dimer	62.1		100		100	
V57D	Monomer	56.1	1.01	6.1	1.12	0	3.18
	Dimer	43.9		93.9		100	
V57N	Monomer	66.5	1.26	15.3	1.08	0	2.56
	Dimer	33.5		84.7		100	
V57G	Monomer	72.4	1.06	24.6	1.00	0	2.15
	Dimer	27.6		75.4		100

However, the exposure of HCC samples to synchrotron radiation for 100 ms resulted in the dimer fraction dominating all samples studied. The maximum dimer content (100%) was observed for the L68V sample, the lowest content (75.4%) was observed for the V57G variant, and the dimer content was 79.9% in the wt-HCC sample. Exposures over 200 ms in all samples led to almost complete oligomerization of the protein (loss of monomer and dimer fractions).

A comparison of experimental SAXS data from wild-type HCC separated using the MCR-ALS procedure with theoretical scattering curves calculated from the atomic structures of monomeric (PDB code: 3GAX) and dimeric (PDB code: 1TIJ) human cystatin C variants is presented in Fig. 4. This example clearly shows the high similarity of separated SAXS data for the monomer and dimer to the theoretical scattering curves calculated from the atomic structures. These provide clear evidence of domain swapping in HCC molecules induced by synchrotron irradiation.

**Figure 6:**
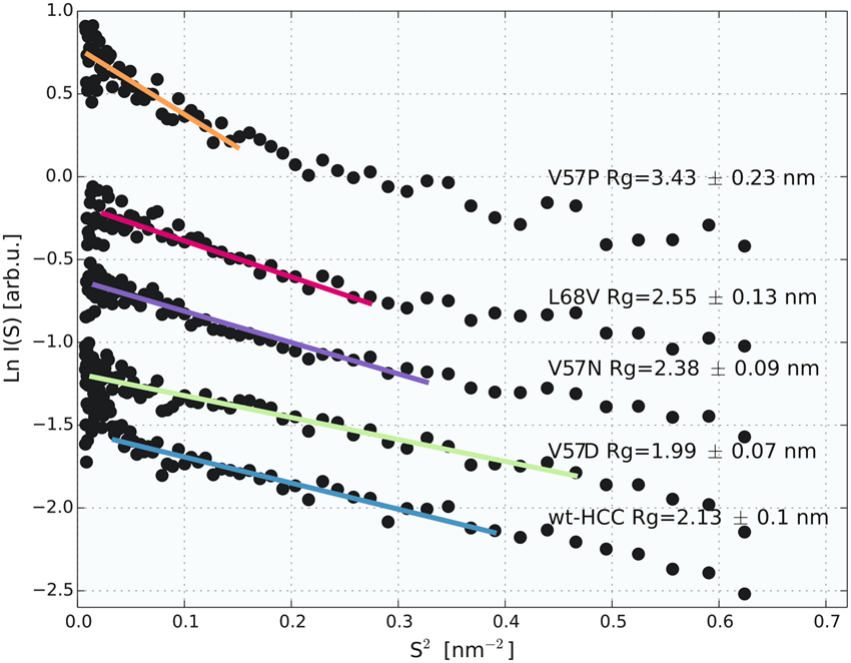
Guinier plot of SANS data recorded for wild-type HCC and its variants.

To independently verify the monodispersity (presence of monomeric form) of non-irradiated cystatin C samples and variants, a series of SANS measurements was performed, and the results are summarized in Fig. 6 in the form of a Guinier plot. The *R*_*g*_ values calculated from the SANS data by fitting to the Guinier equation were similar to those obtained from the 50-ms TR-SAXS measurements.

*R*_*g*_ values that unambiguously indicated the dominant monomeric character of the sample were obtained for wt-HCC (*R*_*g*_ = 2.1 ± 0.1 nm), the V57D variant (*R*_*g*_ = 1.99 ± 0.07 nm) and the V57N variant (*R*_*g*_ = 2.38 ± 0.09 nm). A slightly higher *R*_*g*_ value was noted for the L68V variant (*R*_*g*_ = 2.55 ± 0.13 nm), which as previously noted, had a slightly higher propensity to dimerize than wt-HCC.

As in the TR-SAXS data, the V57P variant exhibited oligomerization in the SANS experiment. The *R*_*g*_ for this variant (3.43 ± 0.23 nm) estimated from SANS data deviates significantly from the *R*_*g*_ values of other HCC variants. The same samples (protein solutions after SANS measurements) were subsequently used to measure diffusion coefficients and hydrodynamic radii via NMR diffusiometry. However, due to the low concentration of the protein in solution, the SANS and diffusiometry measurements were not performed for the V57G variant.

**Figure 7:**
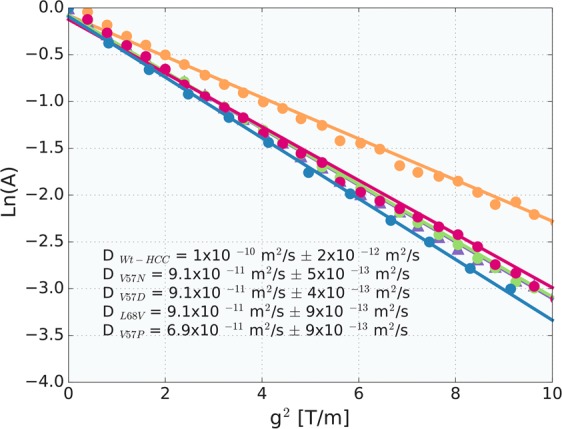
The natural logarithm of the ^1^H NMR spin-echo signal attenuation, ln(A) = ln(*M*(*g*)/*M*(*g* = 0)), versus the amplitude of the magnetic field gradient *g*, where *M*(*g*) values are the signal amplitudes obtained for the region between 1 and 4 ppm. The data points correspond to wt-HCC and HCC variant samples.

The hydrodynamic and diffusion parameters obtained from NMR diffusion measurements are summarized in Table 2. The dependence of ln(*A*) vs *g*^2^ extracted from the 1–4 ppm region in the NMR spectra are shown in Fig. 7. All cystatin variants are in the monomeric state, except V57P, which is a mixture of oligomers. The average value of the diffusion coefficient was 6.90 × 10^−11^ ± 9 × 10^−13^ m^2^/s. The stage of oligomerization was calculated by dividing the selected solution-state hydrodynamic volume by the volume obtained for a wild-type cystatin C molecule (reference sample). Accordingly, we obtained hydrodynamic volume ratios and estimated average aggregation numbers. Higher *V*_*h*_*/V*_*h*_(wt-HCC) ratios indicate that L68V, V57D and V57N cystatin variants occupy slightly higher hydrodynamic volumes.

**Table 2:** Analysis of the hydrodynamic radii, *R*_*h*_, and hydrodynamic volumes, *V*_*h*_.

HCC variant	*D* [m^2^ s^−1^]	*R*_*h*_ [nm]	${{\boldsymbol{R}}}_{{\boldsymbol{g}}}^{{\boldsymbol{\ast }}}$ [nm]	Vol [nm^3^]	*V*_*h*_*/V*_*h*_(wt-HCC)
wt-HCC	1.00 × 10^−10^ ± 2 × 10^−12^	2.21	1.71	45.21	1.00
L68V	9.10 × 10^−11^ ± 9 × 10^−13^	2.42	1.87	59.36	1.31
V57D	9.10 × 10^−11^ ± 4 × 10^−13^	2.39	1.85	57.18	1.26
V57N	9.10 × 10^−11^ ± 5 × 10^−13^	2.39	1.85	57.18	1.26
V57P	6.90 × 10^−11^ ± 9 × 10^−13^	3.19	2.47	135.97	3.01

The ratios were calculated with respect to the diffusion coefficient obtained for wt-HCC. ${{\boldsymbol{R}}}_{{\boldsymbol{g}}}^{{\boldsymbol{\ast }}}$ - value of *R*_*g*_ calculated using the formula *R*_*g*_ = 0.775 *R*_*h*_, valid for spherical proteins^49^.

We assume that the degree of compaction has changed, affecting the solution-state hydrodynamic radii^41^. Additional evidence of aggregation can be observed in the 8–10 ppm region (V57P), where the signal vanished due to an enhanced transverse relaxation time caused by oligomerization (see Fig. S6 in the Supplementary Materials).

**Figure 8:**
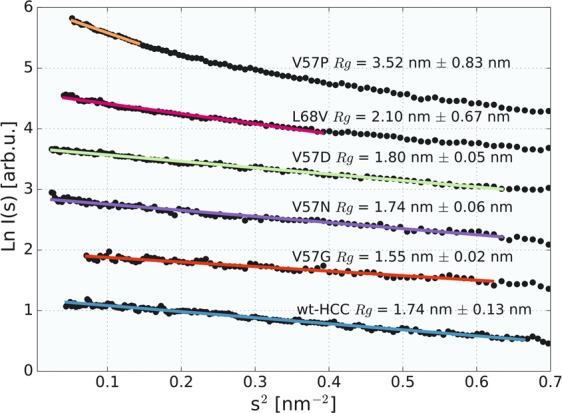
Guinier plot of SAXS data recorded for wild-type HCC and its variants. The experiment was performed using a laboratory SAXS system and GaKα radiation, with an exposition time of 150 min.

For all HCC samples studied, additional SAXS experiments were also conducted under conditions designed to minimize (or effectively eliminate) the potential for radiation damage by using an X-ray source with a different wavelength (GaKα *λ* = 0.134 nm) and significantly lower flux (2 × 10^8^ photons/s) than that in the TR-SAXS experiment. In this experiment, a laboratory SAXS system with a similar setup to the one utilized for synchrotron measurements was used (equipped with a PILATUS3R 1 M detector, covering a similar *s*-vector range). The results of these experiments are shown in Fig. 8 (and Fig. S7 in the Supplementary Materials).

In this experiment, we wanted to assess the impact of gentle irradiation on the monodispersity of the studied samples and to confirm that the increase in dimer content during the TR-SAXS experiment was closely related to the impact of irradiation by the synchrotron beam.

*R*_*g*_ values calculated for all samples (see Fig. 8) in this experiment were slightly lower than those obtained for 50-ms exposure in TR-SAXS, indicating an even higher monomer content. In addition, the contribution of the HCC monomer fraction in the scattering curve calculated by OLIGOMER (see Table S3) was significantly higher than that in the TR-SAXS experiment, as shown in Table 1. The V57G sample was almost completely monomeric (97.9% monomer content, *R*_*g*_ = 1.55 ± 0.02 nm), while the monomer content ranged from 79.8% (V57D, *R*_*g*_ = 1.8 ± 0.05 nm) to 85.5% (wt-HCC, *R*_*g*_ = 1.74 ± 0.13 nm) for the other samples. Only sample L68V showed a 1:1 monomer-dimer ratio, and the V57P sample was highly oligomerized, as in the TR-SAXS experiment. Generally, the data obtained using a low-flux X-ray source were in very good agreement with the SANS and diffusion results.

## Discussion

Different mechanisms for the radiation-induced domain swapping of wild-type cystatin C are possible. The amino acid sequence of the single polypeptide chain contains four cysteine residues, forming two disulfide bridges, C73-C83 and C97-C117, that stabilize the C-terminal domain of cystatin C^42^. These amino acid residues may be prone to radiolysis, but this effect might be only one of the factors that triggers domain swapping in the first stages of irradiation. A second mechanism might be associated with weakening of the hydrogen bonding scheme that stabilizes the protein secondary structure. A significant increase in HCC dimer content is clearly visible as a result of domain swapping. Comparing the theoretical scattering curves for monomeric and dimeric HCC based on the crystallographic data with separated SAXS data revealed substantial similarity between the structures present in solution and the known HCC structures in the crystal. The domain swapping mechanism initiated by irradiation may also be involved in further oligomerization; however, we cannot exclude the possibility that disulfide bridges, potentially disrupted by radiolysis, participate in oligomerization as well.

Importantly, domain swapping is not always required for dimerization or oligomerization, as has been demonstrated in studies of monellin and stefin B (cystatin B), two proteins of the cystatin superfamily. Although monellin has a structural motif similar to that of cystatin C and is usually present as a non-covalent dimer, it has not been possible to show that monellin dimerizes or oligomerizes by the domain swapping mechanism. This problem has been studied by Mascarenhas and Gosavi (2017), who compared dimerized stefin B with monellin^43^.

In the case of HCC, domain swapping has been proposed as the mechanism responsible for oligomer and fibril formation. Recently, HCC was shown to form oligomers without the formation of domain-swapped dimers^10^. These experiments were conducted at acidic pH, far from physiological values. In our case, X-ray-induced HCC oligomerization clearly involves the formation of dimers via domain swapping. Our experiments were conducted under near-physiological conditions at neutral pH and without denaturing agents promoting non-native conformations. As outline above, X-ray radiation may destabilize disulfide bonds or hydrogen bond networks, but within the first 50–150 ms, the monomeric form of HCC is present with a well-preserved tertiary structure. At the HCC concentration used, molecular crowding likely leads to the formation of more energetically favourable dimers, as was shown for HCC fibrillation *in vitro*^8^. To our knowledge, these findings represent the first structural evidence for the transition from the monomeric to oligomeric form of HCC via the formation of a domain-swapped dimer, strongly supporting the hypothesis that dimer formation is required for HCC oligomerization and amyloid fibril formation.

Several crystal structures of wt-HCC and its variants have been published^4,6,30,44^. The native form of HCC has been crystallized in two polymorphic crystal forms (cubic and tetragonal)^45^. In both crystal structures, HCC molecules exist as dimers but dimerize in the swing angle of one domain with respect to the other^4,6^. This effect indicates the high conformational flexibility of this protein. The domain-swapping dimerization observed in the crystal state most likely arises from the crowding effect during crystallization and must be associated with a higher thermodynamic stability of the dimer than of the monomer. Environmental factors such as high-energy radiation are unlikely to change the state of the protein in the crystal lattice. However, the situation changes for proteins in solution, where such influences cannot be ruled out. Indeed, we observed this for HCC.

Looking for similar phenomena induced by irradiation in other proteins, a similar domain swapping induced by UV radiation has been observed for chicken egg white lysozyme^46^. However, the irradiation of this protein lasted much longer (up to 96 h), and the radiation energy was significantly lower (*λ* = 285 nm, 4.35 eV). As a result of irradiation, the disruption of disulfide bridges stabilizing the lysozyme tertiary structure, followed by domain swapping of the C-terminal fragment and the formation of fibrils, were observed in this experiment. However, Xie *et al*. suggested that lysozyme molecules show a globular structure (native-like conformation) in fibrils after irradiation and still have catalytic activity^46^. A similar mechanism of reproducing native-like subunits in dimers after propagated domain swapping or the formation of higher-order oligomers and amyloid fibrils has also been postulated for HCC^8^. However, the formation of domain-swapped HCC oligomers or fibrils, as described to date, has been induced only by acidic environments, temperature and shear forces. As far as we know, the present study is the first to demonstrate conformational changes (domain swapping) induced by X-rays, not only in HCC but in any protein.

Notably, high-energy radiation is also able to induce changes in the secondary structure of proteins. In bovine serum albumin (BSA) exposed to gamma radiation (5 kGy dose), significant changes in the composition of secondary structure elements were observed after irradiation^47^. In this case, the alpha helical content decreased by 8% following irradiation, and the content of beta elements (sheets and turns) increased by approximately 3%. Whereas X-ray-induced changes in proteins are well documented in the case of protein crystallography, SAXS studies have primarily focused on preventing radiation damage^23,48^. In our case, X-ray radiation had a direct effect on the structure of HCC, leading to the formation of domain-swapped dimers, and not to non-specific aggregation. Thus, for many structurally labile proteins, SAXS results may lead to misleading conclusions about the conformations of the studied proteins in the absence of sufficiently careful data analysis. On the other hand, a very bright source of synchrotron radiation combined with a fast-readout detector may be used to detect specific structural changes and even discrete transient states of protein molecules.

## Conclusions

We have shown that even short exposure of HCC molecules to a strong X-ray beam can induce domain swapping, which in HCC is crucial for the formation of oligomers and amyloid deposits. At present, it is not possible to estimate the critical value of X-ray dose that may cause this type of conformational changes in tissues; however, one should take into account that the human body is exposed to high-energy radiation during X-ray tomography or radiotherapy. Therefore, further studies of radiation-induced conformational changes, not only for human cystatin C but also for other amyloidogenic proteins that have been observed to undergo domain swapping, are needed to address this problem in more detail.

## Supplementary information

Supporting information for: The domain swapping of human cystatin C induced by synchrotron radiation
