# Supplementary material for: The domain swapping of human cystatin C induced by synchrotron radiation
Source: Sci Rep. 2019 Jun 12;9:8548. doi: 10.1038/s41598-019-44811-1 (PMC6561922; doi:10.1038/s41598-019-44811-1)
Supplement: Supplementary file 1 — Supporting information for: The domain swapping of human cystatin C induced by synchrotron radiation [file 41598_2019_44811_MOESM1_ESM.pdf]

Supporting information for: The domain swapping of human cystatin C induced by synchrotron radiation

Michał Taube,[†,‡] Zuzanna Pietralik,[†] Aneta Szymańska,[¶] Kosma Szutkowski,[†,§] Daniel Clemens,[‖] Anders Grubb,[⊥] and Maciej Kozak[*,†,‡]

†*Department of Macromolecular Physics, Adam Mickiewicz University in Poznań, Uniwersytetu Poznańskiego 2, 61-614 Poznań, Poland*

‡*Joint Laboratory for SAXS Studies, Adam Mickiewicz University in Poznań, Uniwersytetu Poznańskiego 2, 61-614 Poznań, Poland*

¶*Faculty of Chemistry, University of Gdańsk, Wita Stwosza 63, 80-308 Gdańsk, Poland*

§*NanoBioMedical Centre at Adam Mickiewicz University in Poznań, Wszechnicy Piastowskiej 3, 61-614 Poznań, Poland*

‖*Helmholtz-Zentrum Berlin für Materialien und Energie Lise-Meitner-Campus Hahn-Meitner-Platz 1, 14109 Berlin, Germany*

⊥*Department of Clinical Chemistry, Lund University Hospital, S-22185 Lund, Sweden*

E-mail: mkozak@amu.edu.pl

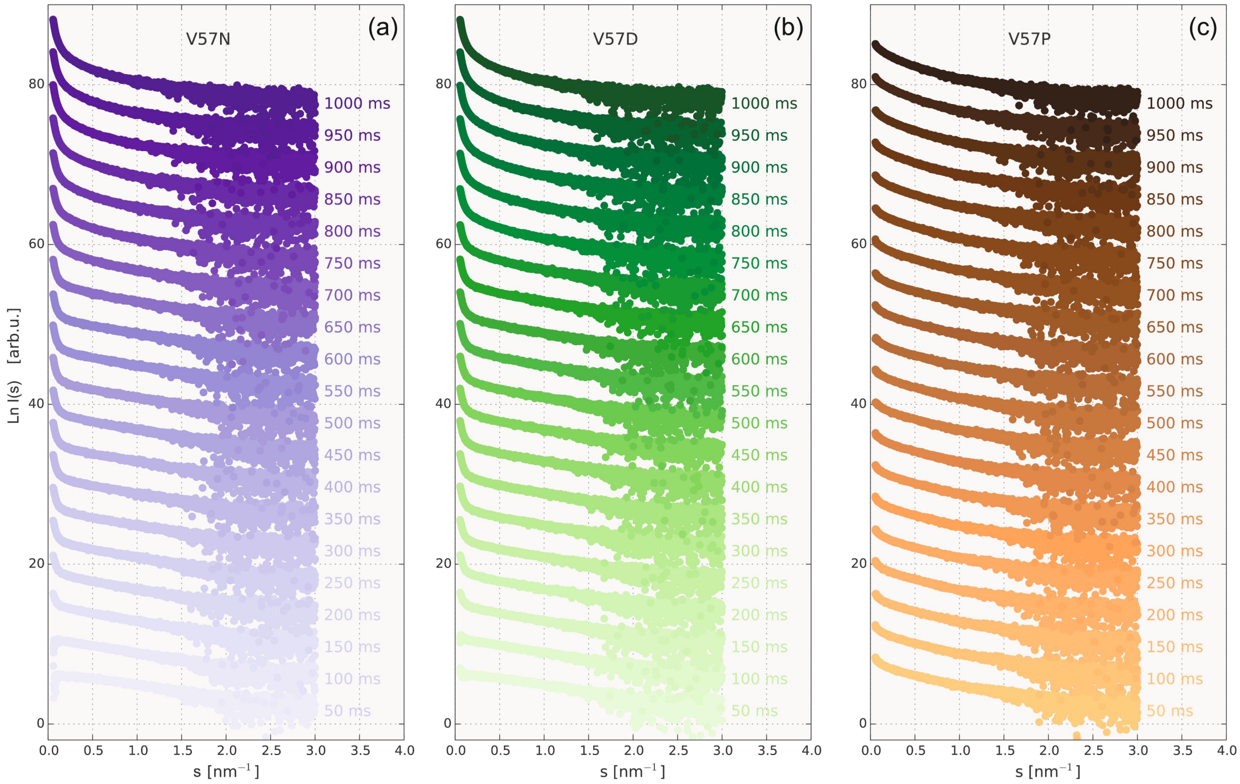

Figure S1: SAXS data (frames 1-20) collected for human cystatin C variants: V57N (a), V57D (b) and V57P (c). The data were shifted for clarity.

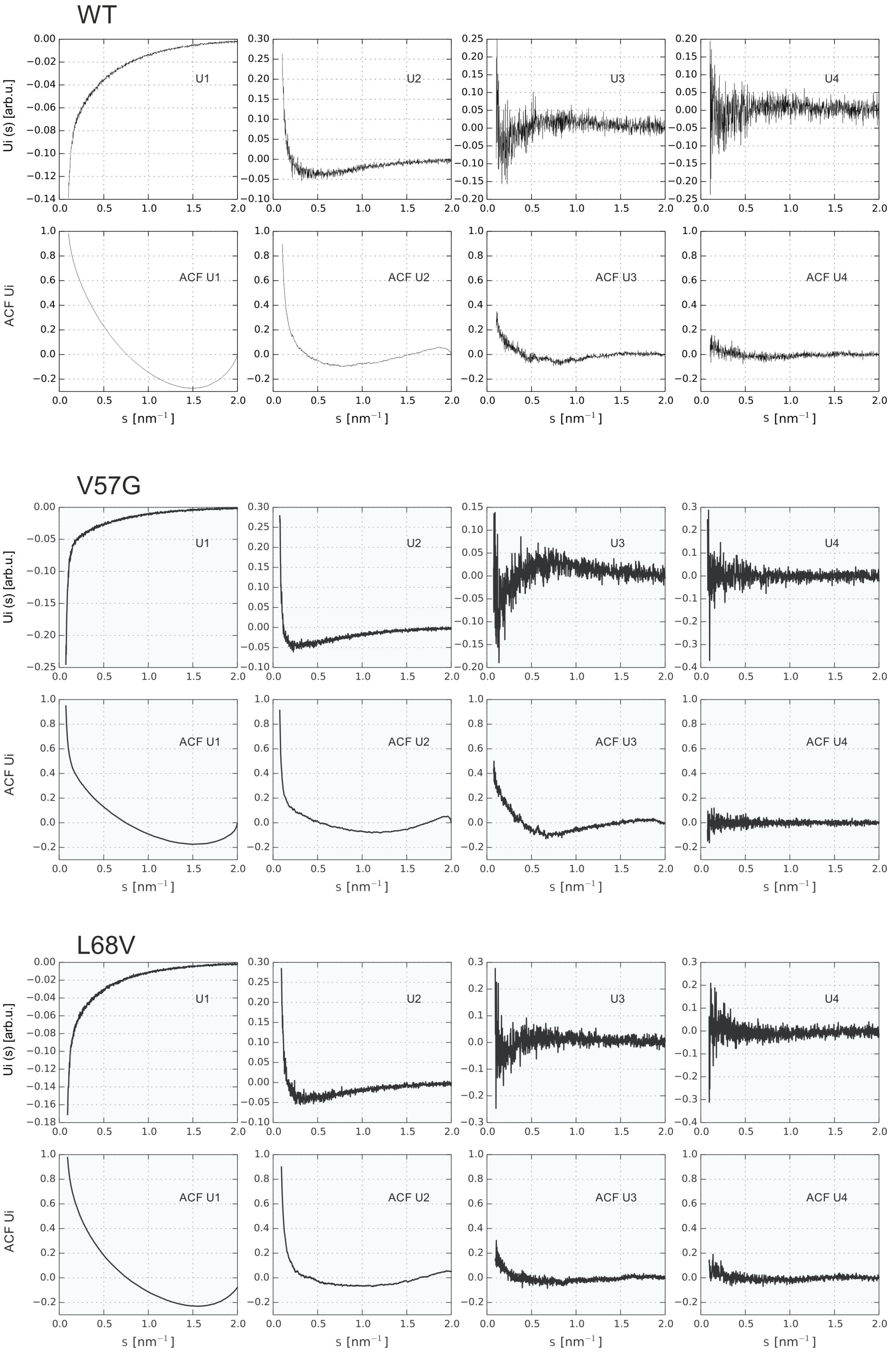

Figure S2: SVD analysis of frames 1-5 (50-250 ms) from TR-SAXS data for the wild type human cystatin C and HCC variants V57G and L68V. In each graph, first row represents four eigenvectors U_i and second its corresponding autocorrelation function ACF U_i.

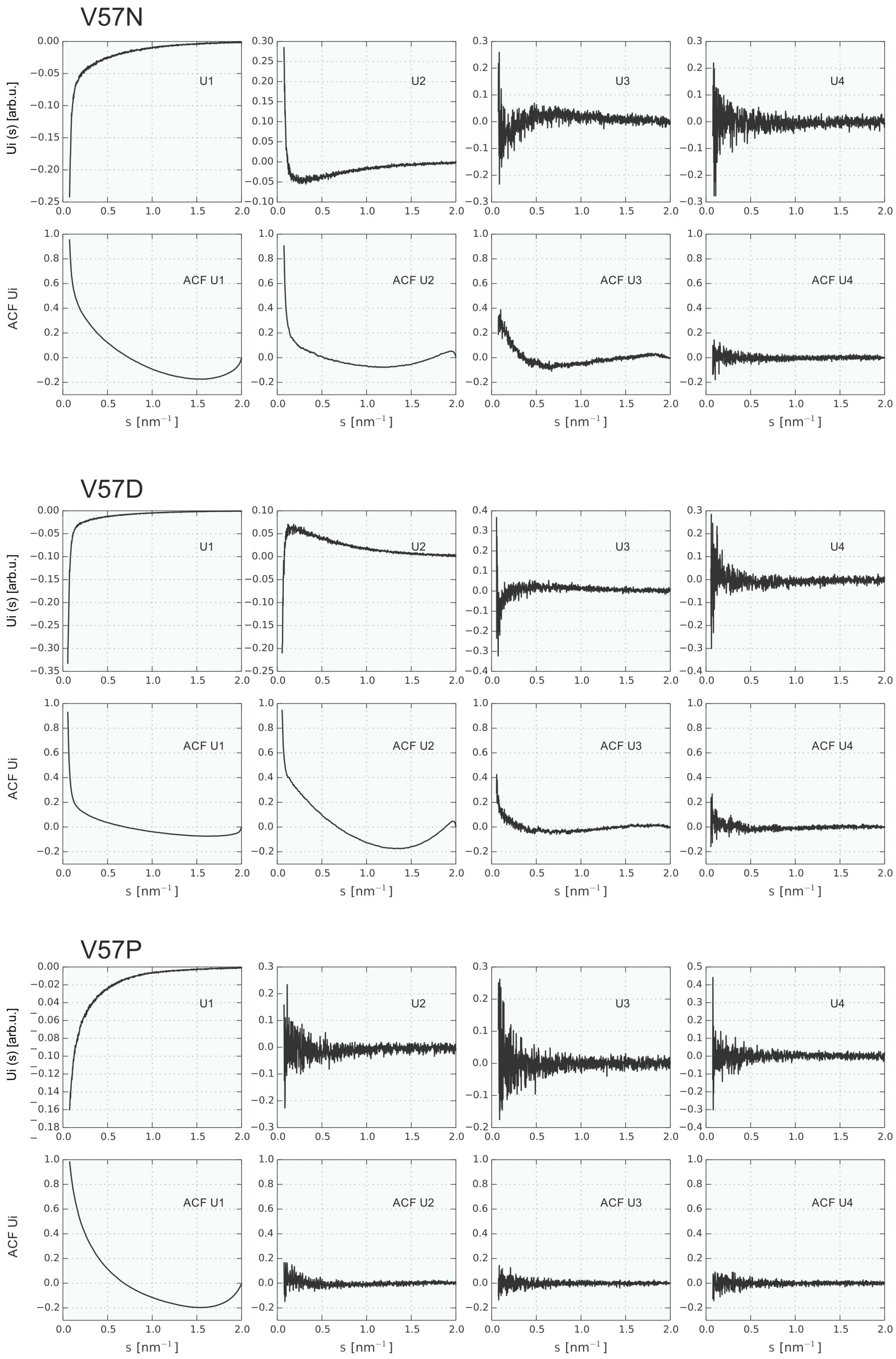

Figure S3: SVD analysis of frames 1-5 (50-250 ms) from TR-SAXS data for human cystatin variants: V57N, V57D and V57P. In each graph, first row represents four eigenvectors U_i and second its corresponding autocorrelation function ACF U_i.

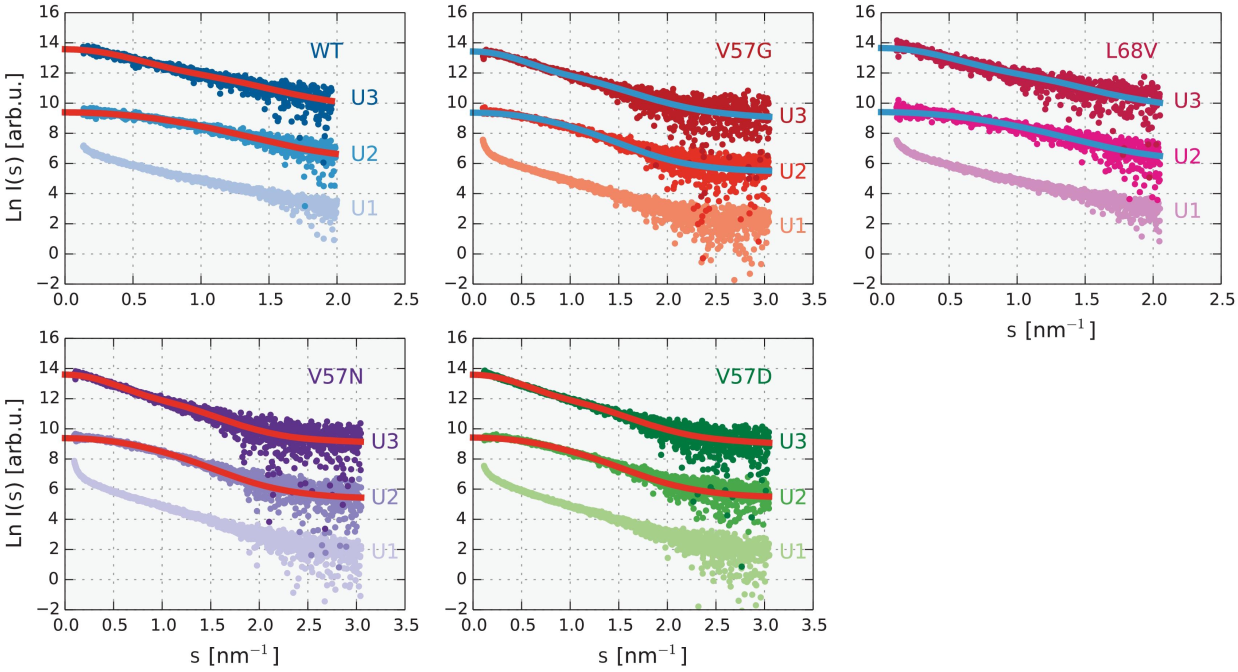

Figure S4: MCR-ALS analysis of SAXS data collected for wild type human cystatin C and its variants: V57G, L68V, V57N, V57D. In each plot reconstructed curves for three SVD detected species: U1-U3 are presented, together with fits of U1 to the stab1-HCC monomer structure (PDB code: 3GAX) and U2 to the structure of wt-HCC dimer (PDB code: 1TIJ). The data were shifted for clarity.

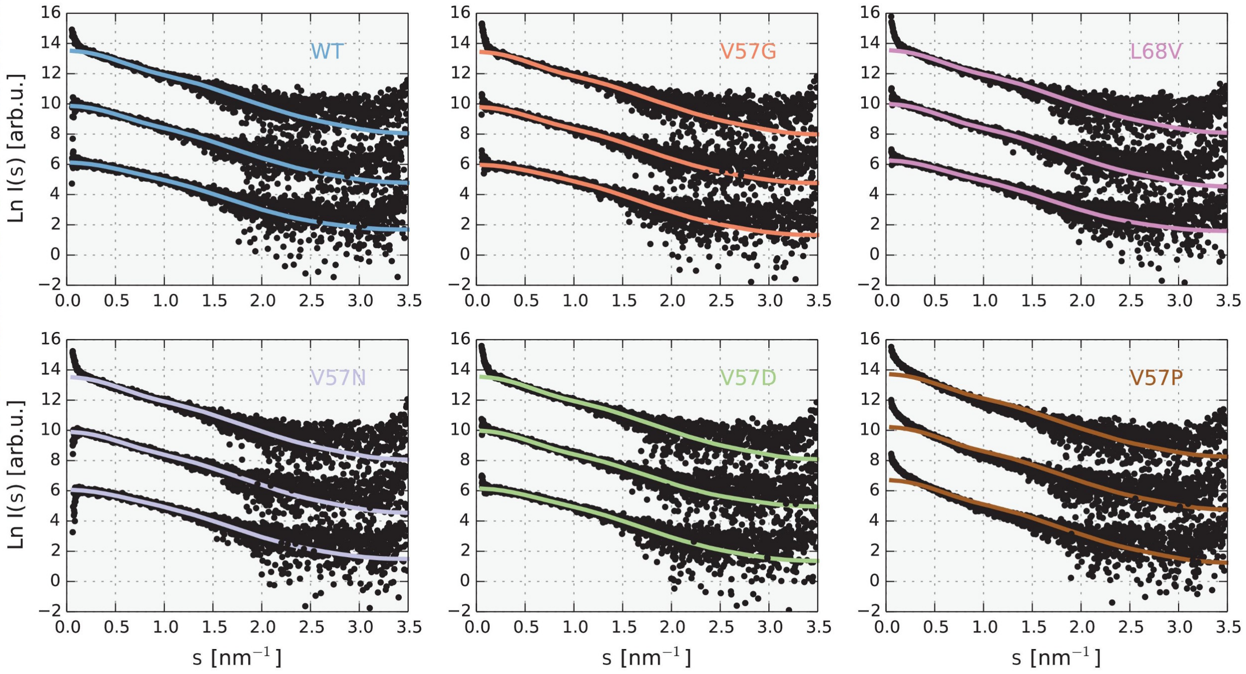

Figure S5: The fits of TR-SAXS data (expositions: 50, 100 and 150 ms), recorded for wild type human cystatin C and its variants, to the monomer (PDB code: 3GAX) and dimer (PDB code:1TIJ) structures of human cystatin C. The fits were calculated using OLIGOMER.[S1] The data were shifted for clarity.

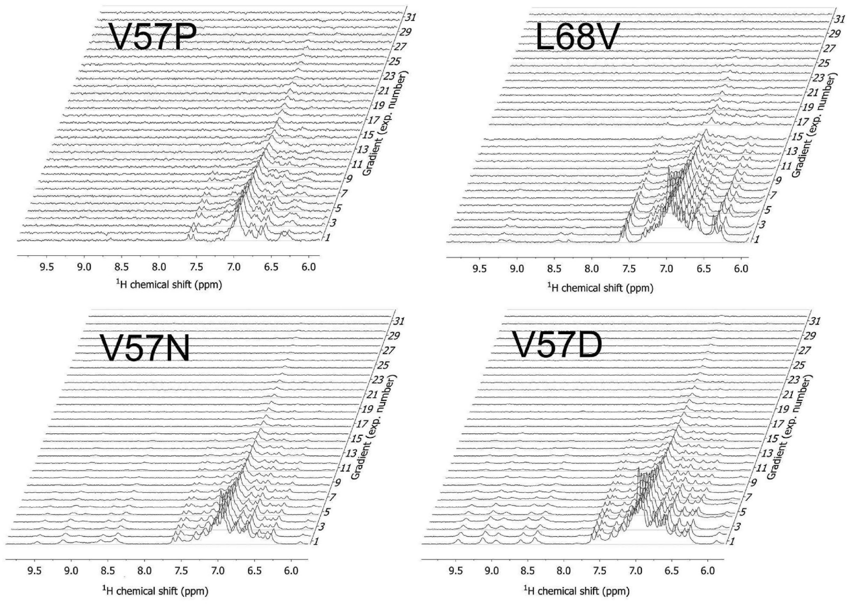

Figure S6: The signal attenuation in the 6-10 ppm region of the diffusion weighted ^{1}H-NMR spectra of human cystatin C variants: V57P, L68V, V57N, V57D.

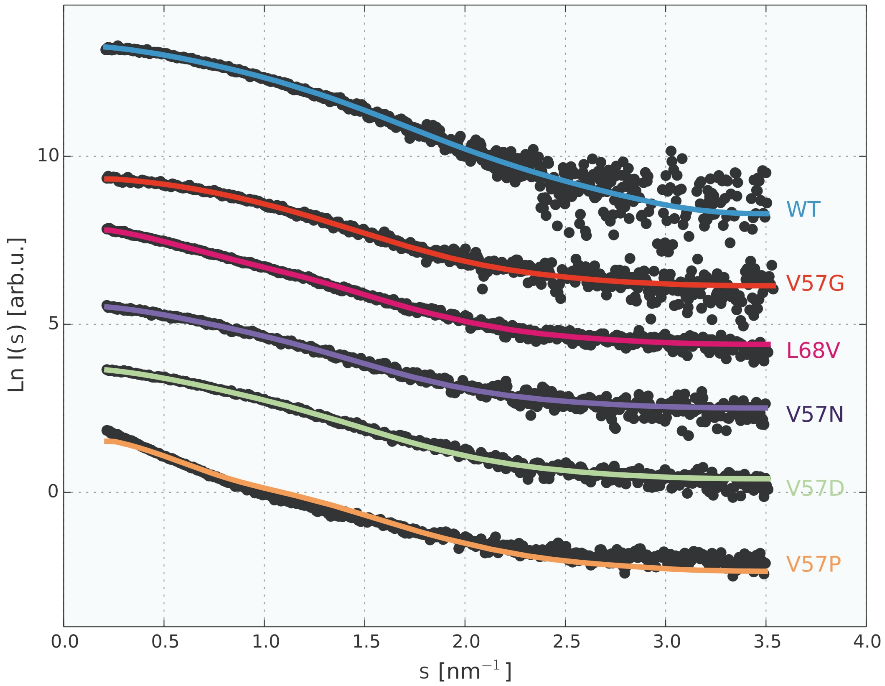

Figure S7: The fits of SAXS data recorded using the laboratory GaK_α X-ray source for wild type human cystatin C and its variants, to the monomer (PDB code: 3GAX) and dimer (PDB code:1TIJ) structures of human cystatin C. The fits were calculated using OLIGOMER. The data were shifted for clarity.

cystatin C variant	50 ms	100 ms	150 ms	200 ms	250 ms	300 ms	350 ms
wt-HCC	2.16 nm±0.18 nm	3.01 nm±0.24 nm	3.57 nm±0.12 nm	4.08 nm±0.13 nm	4.88 nm±0.14 nm	5.34 nm±0.21 nm	6.27 nm±0.28 nm
V57G	2.03 nm±0.16nm	2.84 nm±0.29 nm	3.50 nm±0.06 nm	4.27 nm±0.1 nm	4.54 nm ±0.12 nm	5.42 nm±0.2 nm	5.6 nm±0.17 nm
L68V	2.45nm±0.18 nm	3.41 nm±0.22 nm	3.84 nm±0.07 nm	4.82 nm±0.12 nm	5.21 nm±0.21 nm	n.d.	n.d.
V57N	2.07 nm±0.14 nm	3.08 nm±0.33 nm	3.48 nm±0.06 nm	4.02 nm±0.06 nm	4.38 nm±0.11 nm	4.75 nm±0.13 nm	5.11 nm±0.19 nm
V57D	2.16 nm±0.12 nm	3.00 nm±0.12 nm	3.86 nm±0.06 nm	4.39 nm±0.08 nm	4.81 nm±0.09 nm	5.55 nm±0.18 nm	n.d.
V57P	4.63 nm±0.21 nm	4.76 nm±0.23 nm	4.70 nm±0.22 nm	4.84 nm±0.21 nm	5.61 nm±0.22 nm	5.05 nm±0.43 nm	5.03 nm±0.3 nm

Table S1: Rg values for the wild type human cystatin C and its variants estimated on the basis of the TR-SAXS data. n.d. - not determined.

Table S2: χ^2 value for the fits the structures of monomeric (PDB code: 3GAX) and dimeric (PDB code: 1TIJ) forms of human cystatin C to the reconstructed species U1 and U2 from the MCR-ALS analysis.

Cystatin C variant	U1 monomer fit χ^2	U2 dimer fit χ^2
wt-HCC	1.64	1.72
V57G	2.17	1.73
L68V	1.74	1.85
V57N	2.14	1.77
V57D	2.41	2.34

Table S3: Monomer and dimer fractions of HCC variants calculated using OLIGOMER for laboratory SAXS data.

Cystatin C variant	Monomer fraction	Dimer fraction	χ^2
wt-HCC	85.5 %	14.5 %	1.02
V57G	97.9 %	2.1 %	0.745
L68V	50.1 %	49.9 %	0.83
V57N	81.2 %	18.8 %	0.86
V57D	79.9 %	20.1 %	0.84
V57P	0 %	100 %	14.04

References

(S1) Konarev, P. V.; Volkov, V. V.; Sokolova, A. V.; Koch, M. H. J.; Svergun, D. I. *J. Appl. Crystallogr.* **2003**, *36*, 1277–1282.
